# Critical Predictors for the Early Detection of Conversion From Unipolar Major Depressive Disorder to Bipolar Disorder: Nationwide Population-Based Retrospective Cohort Study

**DOI:** 10.2196/14278

**Published:** 2020-04-03

**Authors:** Ya-Han Hu, Kuanchin Chen, I-Chiu Chang, Cheng-Che Shen

**Affiliations:** 1 Center for Innovative Research on Aging Society National Chung Cheng University Chiayi County Taiwan; 2 MOST AI Biomedical Research Center National Cheng Kung University Tainan City Taiwan; 3 Department of Information Management National Central University Taoyuan City Taiwan; 4 Department of Business Information Systems Western Michigan University Kalamazoo, MI United States; 5 Department of Information Management and Institute of Healthcare Information Management National Chung Cheng University Chiayi County Taiwan; 6 Department of Psychiatry, Chiayi Branch Taichung Veterans General Hospital Chiayi City Taiwan; 7 School of Medicine National Yang-Ming University Taipei Taiwan

**Keywords:** major depressive disorder, bipolar disorder, National Health Insurance Database, data mining, classification and regression tree

## Abstract

**Background:**

Unipolar major depressive disorder (MDD) and bipolar disorder are two major mood disorders. The two disorders have different treatment strategies and prognoses. However, bipolar disorder may begin with depression and could be diagnosed as MDD in the initial stage, which may later contribute to treatment failure. Previous studies indicated that a high proportion of patients diagnosed with MDD will develop bipolar disorder over time. This kind of hidden bipolar disorder may contribute to the treatment resistance observed in patients with MDD.

**Objective:**

In this population-based study, our aim was to investigate the rate and risk factors of a diagnostic change from unipolar MDD to bipolar disorder during a 10-year follow-up. Furthermore, a risk stratification model was developed for MDD-to-bipolar disorder conversion.

**Methods:**

We conducted a retrospective cohort study involving patients who were newly diagnosed with MDD between January 1, 2000, and December 31, 2004, by using the Taiwan National Health Insurance Research Database. All patients with depression were observed until (1) diagnosis of bipolar disorder by a psychiatrist, (2) death, or (3) December 31, 2013. All patients with depression were divided into the following two groups, according to whether bipolar disorder was diagnosed during the follow-up period: converted group and nonconverted group. Six groups of variables within the first 6 months of enrollment, including personal characteristics, physical comorbidities, psychiatric comorbidities, health care usage behaviors, disorder severity, and psychotropic use, were extracted and were included in a classiﬁcation and regression tree (CART) analysis to generate a risk stratification model for MDD-to-bipolar disorder conversion.

**Results:**

Our study enrolled 2820 patients with MDD. During the follow-up period, 536 patients were diagnosed with bipolar disorder (conversion rate=19.0%). The CART method identified five variables (kinds of antipsychotics used within the first 6 months of enrollment, kinds of antidepressants used within the first 6 months of enrollment, total psychiatric outpatient visits, kinds of benzodiazepines used within one visit, and use of mood stabilizers) as significant predictors of the risk of bipolar disorder conversion. This risk CART was able to stratify patients into high-, medium-, and low-risk groups with regard to bipolar disorder conversion. In the high-risk group, 61.5%-100% of patients with depression eventually developed bipolar disorder. On the other hand, in the low-risk group, only 6.4%-14.3% of patients with depression developed bipolar disorder.

**Conclusions:**

The CART method identified five variables as significant predictors of bipolar disorder conversion. In a simple two- to four-step process, these variables permit the identification of patients with low, intermediate, or high risk of bipolar disorder conversion. The developed model can be applied to routine clinical practice for the early diagnosis of bipolar disorder.

## Introduction

Unipolar major depressive disorder (MDD) and bipolar disorder are two common mood disorders in psychiatry. Both disorders are associated with severe functional impairment and disability [1-6], but they have different clinical courses, treatment strategies, and prognoses. However, the course of bipolar disorder may begin with depression, and it could be incorrectly diagnosed as MDD in the initial stage [7,8]. As previous studies have shown [9-28], a high proportion of patients diagnosed with MDD will develop bipolar disorder (0%-37.5%) over time. Furthermore, this kind of hidden bipolar disorder may contribute to the treatment resistance observed in unipolar depression [15,29]. Previous results showed that more than 50% of people with treatment-resistant unipolar depression were subsequently diagnosed with occult bipolar disorder when reappraised during the follow-up period [29,30]. Furthermore, the use of antidepressants for the acute and maintenance treatment of bipolar depression is controversial because of concerns that these drugs are not effective and may harm patients by causing a switch from depression to mania [31-33]. With this knowledge, most doctors may want to avoid using antidepressants as monotherapy for bipolar depression. However, according to the study by Goldberg et al, less than one-half of patients with depression who showed eventual bipolar disorder conversion had received prescriptions for mood stabilizers in any of the follow-up years [21]. Given the therapeutic and prognostic significances of the unipolar-bipolar dichotomy, predicting which patients will show bipolar disorder subsequent to an index diagnosis of MDD is of considerable clinical importance.

Although some studies have investigated the rate of MDD-to-bipolar disorder conversion and the risk factors for a diagnostic change [9-28], their results were inconsistent and sometimes contradictory. For example, the rate of conversion has been reported to be anywhere between 0% and 37.5%, and part of this could be attributed to the limitations in existing studies. First, most of these studies had small samples [9-11,20,21,25,34]. For instance, the study by Rao et al included only 28 patients with depression [25]. Small sample sizes pose challenges to any statistical analysis and cause variability in prediction accuracy. Second, most of these studies included samples enrolled from a single hospital and were not population-based studies, making the epidemiologic generalizability of the findings uncertain. Third, the follow-up duration of some studies might have been too short to detect bipolar disorder conversion in patients with depression. Furthermore, although psychiatric comorbidity is very common in MDD and bipolar disorder, the association of psychiatric comorbidity with bipolar switch has been examined surprisingly little in previous studies. In a recent study, a high bipolar disorder conversion rate was noted in patients with depression who had comorbidities including obsessive-compulsive disorder and social phobia [18]. Whether other physical or psychiatric comorbidities provide additional predictive value for bipolar disorder conversion is worthy of further study. Finally, no previous study has developed a risk stratification model for MDD-to-bipolar disorder conversion. With such an explanatory model in place, patients could be categorized into risk groups, which would allow early interventions and preventive procedures to be formulated. Such a model based on a longitudinal trend backed by data representing the population rather than statistical sampling is important from theoretical and nosological standpoints and may be useful for treatment planning.

Given the therapeutic and prognostic significances of the unipolar-bipolar dichotomy, predicting which patients will show bipolar disorder subsequent to an index diagnosis of MDD is of considerable clinical importance. With small-sample or single-hospital case studies popular in the existing literature, it is difficult to develop such an index with high acceptance across the health care industry. In this population-based study, our aims were three-fold. First, we aimed to investigate the rate of diagnostic change from unipolar MDD to bipolar disorder during a 10-year follow-up using the Taiwan National Health Insurance Research Database (NHIRD). Second, we aimed to develop a risk stratification model using the classiﬁcation and regression tree (CART) technique for MDD-to-bipolar disorder conversion. Third, we aimed to evaluate the performance of prediction models developed with machine learning techniques by using the train-validation-test set split approach.

## Methods

### Data Source

Taiwan has instituted the National Health Insurance (NHI) program, a mandatory single-payer program that offers comprehensive medical care coverage [35]. Moreover, as of 2014, 99.9% of Taiwan’s population was enrolled in this program.

Since 1996, the NHI reimbursement data in Taiwan have been transferred to the National Health Research Institute (NHRI) for further management and organization. In addition, as part of these efforts, the work of the NHRI has resulted in the establishment of a national health care database called the NHIRD, which includes comprehensive information on clinical practice, including patient demographic characteristics, medical expenditure, prescription claims data, surgery codes, treatment codes, and diagnostic codes according to the International Classification of Disease, Ninth Revision, Clinical Modification (ICD-9-CM).

In this study, the Longitudinal Health Insurance Database (LHID) 2000 from 1996 to 2013, which is a dataset released by the NHRI, was used as the data source. The LHID 2000 contains all the original claims data of 1,000,000 beneficiaries enrolled in the year 2000, who were randomly sampled from the year 2000 Registry for Beneficiaries of the NHIRD.

### Ethics Statement

This study was approved by the Institutional Review Board of Taichung Veterans General Hospital (approval number: 2018-07-016AC). As the NHI data set includes deidentified secondary data for research purposes, written consent from the patients for this study was not necessary. Formal written waiver for the requirement of consent was issued by the Institutional Review Board of Taichung Veterans General Hospital.

### Study Population

Using the LHID 2000, we conducted a retrospective cohort study involving patients who were newly diagnosed with MDD between January 1, 2000, and December 31, 2004. MDD was defined according to ICD-9-CM codes 296.2X and 296.3X in ambulatory care expenditure by visit (CD) and inpatient expenditure by admission (DD) files. To ensure diagnostic validity and patient homogeneity, we included patients who were diagnosed only by psychiatrists. We excluded patients who were diagnosed with depressive disorder (ICD-9-CM codes 296.2X, 296.3X, 300.4, and 311.X) from 1996 to 1999 and those who were diagnosed with bipolar disorder (ICD-9-CM codes 296.0, 296.1, 296.4, 296.5, 296.6, 296.7, 296.8, 296.80, and 296.89) before enrolment. In addition, patients who were diagnosed with schizophrenia (ICD-9-CM code 295) were excluded. The index date was deﬁned as the date when an eligible patient with depression was included in our cohort. All patients with depression were observed until (1) diagnosis of bipolar disorder (ICD-9-CM codes 296.0, 296.1, 296.4, 296.5, 296.6, 296.7, 296.8, 296.80, and 296.89) by a psychiatrist, (2) death, or (3) December 31, 2013. All patients with depression were divided into the following two groups, according to whether bipolar disorder was diagnosed during the follow-up period: converted group and nonconverted group.

### Definitions of Research Variables

Factors, including adolescent or early adult age at onset [12,17,19,22], bipolar family history [11,12,19,21,22], loaded pedigrees [11,12], psychosis [11,19,21,22], hypersomnic-retarded phenomenology [11,12], more marked self-reproach and guilt [13], large number of cluster B personality disorder symptoms [18], pharmacologically induced hypomania [11,12], precipitation by childbirth [12], rapid symptom onset [11], higher number of previous episodes [13,23], recurrent admission [13,23], higher rate of functional disruption [17], chronicity of the index episode [19], shorter well intervals [17], severity of MDD [18], history of poor response to antidepressants [15], obsessive-compulsive disorder comorbidity [18], social phobia comorbidity [18], and higher rate of substance abuse [17], have been reported to distinguish converters from nonconverters. Therefore, six groups of variables within the first 6 months of enrollment, including personal characteristics, physical comorbidities, psychiatric comorbidities, health care usage behaviors, disorder severity, and use of psychotropics, were extracted.

Personal characteristics were extracted from registry for beneficiaries (ID) files. We estimated the monthly income according to the patients’ insurance premiums, which are calculated according to the total income of beneficiaries. Monthly income was grouped into low income (monthly income <20,000 New Taiwan Dollar [NTD]), median income (monthly income ≥20,000 NTD but <40,000 NTD), and high income (monthly income ≥40,000 NTD). Urbanization was divided into the following three groups: urban, suburban, and rural. Urbanization and monthly income were used to represent the socioeconomic status. Psychiatric and physical comorbidities were defined according to ICD-9-CM codes in CD and DD files. Disorder severity was defined according to the following two variables: refractory depression and MDD catastrophic illness. Refractory depression was considered when at least two trials of different antidepressants (adequate in terms of dosage and duration) failed to produce a relevant clinical improvement. In our study, we considered participants to have refractory depression if their antidepressant treatment regimen was altered two or more times. An adequate trial was defined as using an antidepressant within its therapeutic dosage range for more than 60 consecutive days [15]. MDD catastrophic illness was defined according to ICD-9-CM codes 296.2X and 296.3X in registry for catastrophic illness patient files. Health care usage behaviors defined in this study included number of total outpatient visits, number of total psychiatric outpatient visits, number of total emergency visits, number of total hospitalizations, number of total psychiatric hospitalizations, number of outpatient visits by month and season, number of psychiatric outpatient visits by month and season, number of emergency visits by month and season, and number of hospitalizations by month and season. The psychotropics surveyed in this study included benzodiazepines, antidepressants, mood stabilizers, and antipsychotics, and information was extracted from details of ambulatory care order and details of inpatient order files. We recorded the kinds of benzodiazepines, antidepressants, mood stabilizers, and antipsychotics that had been used within the first 6 months of enrollment and the maximum kinds of benzodiazepines, antidepressants, mood stabilizers, and antipsychotics that had been administered in one visit.

### Descriptive Statistical Analysis

The chi-square and independent *t*-tests were performed to examine differences in variables between the converted and nonconverted groups.

### Risk Stratification Using the Classiﬁcation and Regression Tree Method

For analyzing variables of interest in converted and nonconverted patients, this study performed CART analysis to generate a risk stratification CART using a complete set of cohort data and variables. The CART method, proposed by Breiman et al, is a well-known machine learning technique [36]. In the field of epidemiology, the CART method has been successfully applied to develop risk stratification models [37,38]. Compared with conventional multivariate statistical methods, such as logistic regression, CART analysis does not require parametric assumptions and can handle highly skewed data. The information extracted by the CART analysis is in the form of if-then rules, which can be easier to apply for bedside assessment and other clinical applications. To simplify the generated risk stratification CART, the minimum number of samples in a leaf node was set to 60. After the CART was built, the bipolar disorder percentage was calculated for each of the leaf nodes in the CART and used to generate the risk stratification model.

### Evaluation of the Prediction Models

Because there were numerous potential independent variables, a number of feature selection and engineering techniques could be performed. First, a correlation-based feature selection (CFS) method could be used to evaluate the correlations among feature subsets to uncover potential collinearity and to assess their predictive power on the response variable [39]. Second, principal component analysis (PCA) is an unsupervised feature engineering technique for dimension reduction, that is, PCA performs linear combination of original independent variables to generate a new set of features in a lower dimensional space. Third, the wrapper method is a feature selection process that measures the usefulness of features according to a user-specific machine learning algorithm.

Four well-known supervised learning techniques, including C4.5 [40], logistic regression (LGR) [41], random forest (RF) [42], and support vector machine (SVM) [43], were used to evaluate the performance of the prediction models.

We partitioned the collected data into fully independent training/validation and testing (ie, holdout) sets. Specifically, two-thirds of patients were randomly included in the training/validation set (1788 patients) to build the prediction models and the remaining one-third of patients were included in the testing set (894 patients) to validate the prediction models [44,45]. In the training/validation set, 267 (14.93%) patients were diagnosed with bipolar disorder, suggesting an imbalanced ratio between the two class labels. To avoid the class imbalance problem, the resample module of Waikato Environment for Knowledge Analysis (Weka) software was employed to under-sample the majority class. As a result, bipolar disorder conversion and nonbipolar disorder conversion cases were adjusted in a 1:1 ratio in the training/validation set. For each training/validation set, the 10-fold cross-validation process was performed, and the mean accuracy, sensitivity, specificity, and area under the curve (AUC) of 10 partitions were calculated.

The model performance metrics, including accuracy, sensitivity, and speciﬁcity, were used in this study because of their widespread adoption and robustness in the ﬁeld of health care [46,47]. In addition, a receiver operating characteristic curve was used to measure the AUC. General rules deﬁned by Hosmer et al [48] were followed to classify the evaluation performance by deﬁning the AUC as follows: excellent, AUC≥0.9; good, 0.9>AUC≥0.8; fair, 0.8>AUC≥0.7; poor, 0.7>AUC≥0.6; and very poor, AUC<0.6.

### Tools for Analysis

Microsoft SQL Server 2005 (Microsoft Corp, Redmond, Washington, USA) was employed for data extraction, computation, linkage, and processing. SPSS (Version 19.0 for Windows; IBM Corp, Armonk, New York, USA) and SAS (Version 9.2; SAS Institute Inc, Cary, North Carolina, USA) were used to perform all statistical analyses. Relationships were considered statistically significant at a *P* value <.05. The simpleCART module in Weka 3.8.2 open-source machine learning software [49] was used to perform the CART analysis. In addition, the CfsSubsetEval module with the BestFirst search algorithm (CFS), the PrincipalComponents module with the Ranker search algorithm (PCA), and the WrapperSubsetEval module with J48 and the BestFirst search algorithm (WrapperJ48) in Weka 3.8.2 were used to perform the feature engineering procedures. In the evaluation of the prediction models, all the selected supervised learning techniques were conducted using the open-source Orange 3.24.0 tool [50].

## Results

### Baseline Data

**Figure 1:**
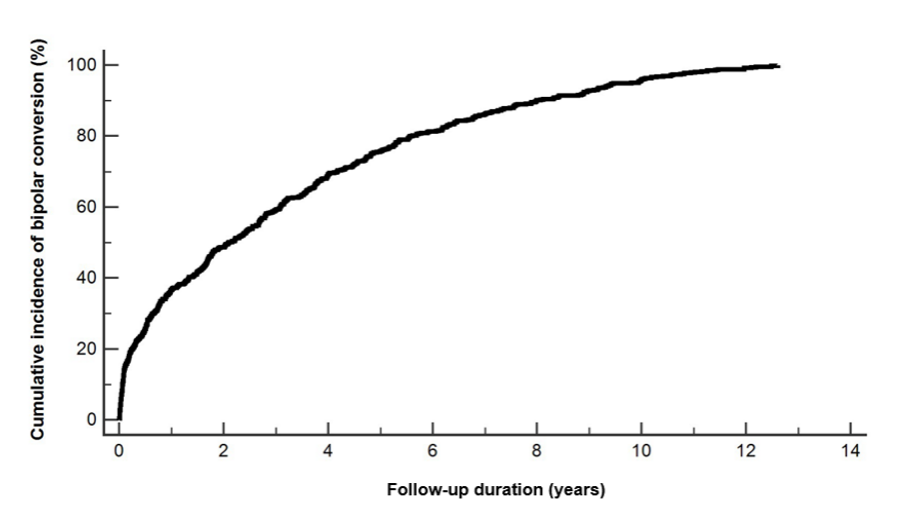
The cumulative incidence of bipolar conversion.

This study enrolled 2820 patients with MDD, among whom 1619 (60.1%) patients were women. The median age at enrollment was 38 years (IQR 26-52 years). During the follow-up period, 536 patients were diagnosed with bipolar disorder (19.0%). The cumulative incidence of bipolar disorder conversion is shown in Figure 1. A total of 138 patients were diagnosed with bipolar disorder within 6 months of enrollment and were excluded. The characteristics in the converted and nonconverted groups are shown in Multimedia Appendix 1. The median age at enrollment was lower in the converted group than in the nonconverted group. The median follow-up duration in the converted group was 2.1 years (IQR 0.5-4.8 years). Furthermore, 178 variables were defined in this study.

### Results of the Classiﬁcation and Regression Tree Analysis

**Figure 2:**
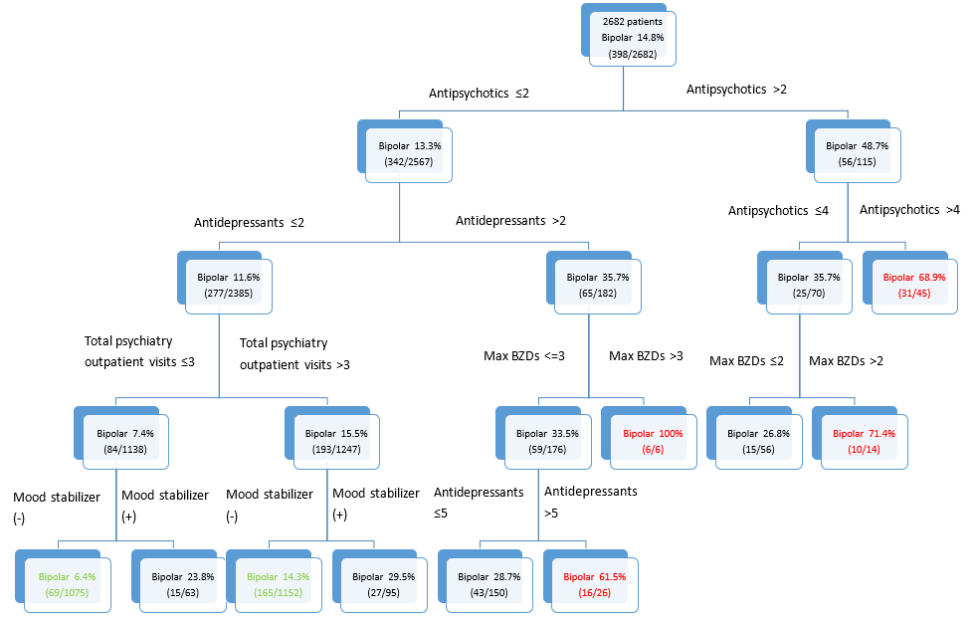
The classiﬁcation and regression tree (CART) risk stratification model for the conversion of bipolar disorder. BZD: benzodiazepine.

By using variables within the first 6 months of enrollment, the decision tree generated through CART analysis is shown in Figure 2. For ease of explanation, we only presented the first four levels of the tree. Among the studied characteristics, the CART method identified the kinds of antipsychotics used as the optimal discriminator between bipolar converters and nonconverters. Other identified characteristics included the kinds of antidepressants used, total psychiatric outpatient visits, kinds of benzodiazepines used within one visit, and use of mood stabilizers. The risk CART was able to stratify patients into high-, medium-, and low-risk groups. In the high-risk group, 61.5%-100% of patients with depression eventually developed bipolar disorder. On the other hand, in the low-risk group, only 6.4%-14.3% of patients with depression developed bipolar disorder. The bipolar disorder conversion OR between the high- and low-risk groups was 188.27 (*P*<.001).

### Performance of the Prediction Models

The results of the evaluation of the performance of the prediction models using the training/validation set are shown in Table 1. According to the average AUC, CFS+RF, CFS+LGR, and PCA+RF were ranked as the top three classifiers. When the average classification accuracy was used as the performance metric instead, CFS+RF, CFS+LGR, and WrapperJ48+RF were ranked as the top three classifiers. Although SVM-based approaches had the highest sensitivity, they exhibited the worst performance regarding specificity. If all performance metrics are taken together, CFS+RF consistently performed very well as compared with the other techniques. The results showed that in the testing set, the accuracy, sensitivity, speciﬁcity, and AUC for CFS+RF were 0.673, 0.695, 0.670, and 0.743, respectively.

Overall, CFS performed the best among the three investigated feature engineering techniques. In CFS, a total of 11 variables were selected, including age, social phobia, obsessive-compulsive disorder, bulimia, total psychiatric outpatient visits within the first 6 months of enrollment, emergency visits (June), outpatient visits (October), kinds of antidepressants used within the first 6 months of enrollment, kinds of antipsychotics used within the first 6 months of enrollment, kinds of benzodiazepines used within the first 6 months of enrollment, and kinds of mood stabilizers used within the first 6 months of enrollment.

**Table 1:** Performance evaluation of prediction models using 10-fold cross-validation.

Feature selection, method		Metric			
		ACC^a^	SEN^b^	SPE^c^	AUC^d^
**CFS^e^**					
	C4.5	0.605	0.577	0.633	0.666
	SVM^f^	0.487	0.914	0.060	0.550
	RF^g^	0.650	0.629	0.670	0.715
	LGR^h^	0.642	0.554	0.730	0.710
**PCA^i^**					
	C4.5	0.582	0.603	0.562	0.644
	SVM	0.493	0.850	0.135	0.543
	RF	0.640	0.652	0.629	0.683
	LGR	0.590	0.521	0.659	0.597
**WrapperJ48**					
	C4.5	0.629	0.479	0.779	0.651
	SVM	0.489	0.835	0.142	0.526
	RF	0.648	0.472	0.824	0.679
	LGR	0.637	0.517	0.757	0.663

^a^ACC: accuracy.

^b^SEN: sensitivity.

^c^SPE: specificity.

^d^AUC: area under the curve.

^e^CFS: correlation-based feature selection.

^f^SVM: support vector machine.

^g^RF: random forest.

^h^LGR: logistic regression.

^i^PCA: principal component analysis.

## Discussion

### Principal Findings

There are several strengths of our study. First, our study design included an unbiased patient selection process. Because participation in the NHI is mandatory and all residents of Taiwan can access health care with low copayment, referral bias is low and follow-up compliance is high. Second, our study was a population-based study and included a large sample from all hospitals in the country. With small-sample or single-hospital studies, which are popular in the existing literature, it is difficult to develop an index with high acceptance across the health care industry. Third, the data used in this study were derived from the NHI system in Taiwan. As an observational database, these data reflect current real-world diagnostic patterns.

The key findings in our study are as follows: (1) the rate of bipolar disorder conversion in patients with MDD was 19%; (2) the median duration of bipolar disorder conversion was 2.1 years (IQR 0.5-4.8 years); (3) the risk of bipolar disorder conversion in patients with MDD can be estimated using the kinds of antipsychotics used, kinds of antidepressants used, total psychiatric outpatient visits, kinds of benzodiazepines used within one visit, and use of mood stabilizers.

Although some studies have investigated the rate of MDD-to-bipolar disorder conversion and the risk factors for diagnostic change [9-28], their results were inconsistent. The reported rates of bipolar disorder conversion vary from 0% to 37.5%. These divergent results may be due to differences in inclusion criteria and the follow-up duration. Previous studies demonstrated that the rate of unipolar-to-bipolar disorder conversion varies across depressive subpopulations [11,12,25,34]. For example, follow-up studies have noted somewhat higher conversion rates in depressed adolescents [11,25,34] than in depressed adults [12,16,23,28]. Furthermore, some studies included inpatient subjects with depression [11,13,16,17,19-21,23,28,34], whereas some studies included outpatient subjects with depression [12,22,26]. The severity of depression in both groups (inpatient and outpatient groups) may differ, which could cause variation in the rates of bipolar disorder conversion. With regard to the duration from the index depressive episode to conversion, a longer follow-up period has been suggested to contribute to more diagnostic switching. The follow-up duration of previous studies ranged from 1 month to 40 years [9-28], which may be one of the major reasons for the different rates of bipolar disorder conversion. In our study, we included all patients with MDD, regardless of age, inpatient status, or outpatient status, and followed up these patients for more than 10 years. The rate of bipolar disorder conversion in patients with MDD was 19%.

With regard to the duration from the first depressive episode to bipolar disorder conversion, in the follow-up study by Winokur and Morrison involving 225 patients with depression from the “Iowa 500” series, nine of the patients showed signs of mania during the course of follow-up from 1 month to 20 years and eight of them had a manic episode within 3 years of their index admission [16]. In the study by Rao and Nammalvar [10], it was reported that 75% of conversions occurred within the first 3 years after the first attack of depression. Dunner et al reported that most switches occur within 18 months from the first depressive episode [9]. In the study by Li et al, a mean time of 1.89-2.98 years for conversion was noted [15]. Similar to previous studies, the results of our work showed that the median duration of MDD-to-bipolar disorder conversion was 2.1 years (IQR 0.5-4.8 years).

Antipsychotics could be augmented with antidepressants in patients with treatment-resistant depression or patients with depression having psychotic features [51]. Our study identified the kinds of antipsychotics used as the optimal discriminator between bipolar converters and nonbipolar converters, and this finding may indicate that bipolar converters have more severe depressive symptoms or psychotic symptoms. This finding is consistent with the results of previous studies showing that psychosis and MDD severity are related to bipolar disorder conversion [11,18,19,21,22]. Furthermore, in the study by Li et al, a history of a poor response to antidepressants was found to be related to bipolar disorder conversion [15]. The authors considered a poor response to antidepressants when the antidepressant treatment regime was altered two or more times. Consistent with these results, our results showed that the kinds of antidepressants used were significant predictors of the risk of bipolar disorder conversion.

Benzodiazepines are safe and effective for relieving common symptoms, such as insomnia, anxiety, and muscle tension [52]. Benzodiazepines are generally not a “core” treatment for mania, but they can rapidly help control certain manic symptoms, such as restlessness, agitation, and insomnia. According to the study by Rizvi et al, with regard to benzodiazepine use, patients with MDD were more likely to be unemployed and have comorbid panic disorder [53]. Their results suggested a more severe functional impairment in benzodiazepine users than in nonusers. On the other hand, Holma et al found that the severity of MDD was related to bipolar disorder conversion [18]. Our study found an association between more kinds of benzodiazepines used within one visit and a higher rate of bipolar disorder conversion. This finding may reflect the association between MDD severity and bipolar disorder conversion.

Models with the abilities to facilitate the early detection of bipolar disorder without sacrificing prediction or classification accuracy have better clinical implications than those without such abilities. Although the performance of our final model using variables within the first 6 months of enrollment was satisfactory, we further conducted a comparative analysis using variables within the first 12 months of enrollment to examine the performance of the prediction model with the same analytical procedures. The results (Multimedia Appendix 2) showed no significant improvement in the AUC between the two datasets (*P*=.09; ie, variables within the first 6 months and the first 12 months of enrollment). This shows two key clinical benefits. First, early detection can be made with data from the first 6 months, which further reduces unnecessary costs and misdiagnosis associated with the traditional approach. Second, it reduces the data volume for clinical analysis without hampering diagnostic accuracy. This empirical evidence adds to clinical practice, as we can now promptly identify high-risk patients for bipolar disorder conversion after collecting data from the first 6 months.

In our study, the results indicated that RF has the highest average AUC in the process of 10-fold cross-validation, and RF use in the testing set showed performance consistent with that in the training/validation set. Many previous studies also found that RF performs better than many standard supervised learning techniques [54-57]. The main advantages of RF are as follows: (1) RF does not involve an assumption that the model has a linear relationship; (2) RF adopts ensemble learning, which forms a strong learner by joining a group of weak learners; and (3) RF iteratively samples data and conducts embedded feature selection to form multiple decision trees. Therefore, RF is recommended as the best classifier owing to its good fault-tolerance ability and low generalization error.

### Contribution to the Literature

Our work adds to the literature in several ways. First, compared with most previous studies based on small or single-hospital samples, our work involved a population-based assessment that offers broader generalizability. The resulting risk classification has wider implications as well. For example, clinical assessments based on the results of small samples are subject to variability owing to possible sampling error, sampling bias, and other common issues that plague small-sample studies. Second, our work is the first study conducted to develop a risk stratification model for MDD-to-bipolar disorder conversion. This model concurrently takes into account demographics, psychiatric comorbidities (ICD-9-CM by the World Health Organization), and usage behavior, providing a holistic view of international health care standards, industry practice, patients, and patient behavior. Finally, our results from studying the longitudinal trend demonstrated that health care usage behaviors and use of psychotropics could be adopted to categorize the risk of bipolar disorder conversion in patients with MDD.

### Contribution to the Industry

The results of our study also have important practical implications. The risk stratification model developed in our study can be easily applied in clinical practice where prediction efficiency is highly valued. For example, a simple questionnaire may be developed according to our findings to check if a patient has the characteristics shown in our risk stratification model. Clinicians could identify patients with bipolar disorder early and arrange appropriate treatment for these patients.

### Limitations and Future Research

Our study is not without limitations. First, information regarding the family history of psychiatric disorders, loaded pedigrees, lifestyle factors, and environmental factors is not included in the NHIRD, all of which might be associated with the risk of bipolar disorder. Second, in studies entailing the use of the NHIRD, it is unclear how diagnostic classification has been conducted, particularly for psychiatric diagnoses. Therefore, the diagnostic accuracy of our study could not be ascertained. Additional studies with patients diagnosed through structured interviews or standard diagnostic criteria should be conducted. Third, the actual severity of depression was not known in our study, and whether this factor influences the risk of conversion warrants further study. Fourth, the duration of the observational period in our study might have been insufficient to detect conversion in certain patients with depression. In addition, different durations of the observational period might be a confounding variable in our study. Future studies with longer and different observational periods are thus required. Fifth, a number of novel feature engineering algorithms have been proposed. Future researchers could consider adopting these techniques to improve the prediction performance. Finally, the accuracy of the prediction model using variables before enrollment could still be improved with variables after enrollment and variables that are not directly collected in the NHIRD, such as lifestyle and severity variables mentioned in the preceding paragraph. Although not the focus of this study, patterns of changes in variables could be further studied to identify changes that have effects on the accuracy of diagnostic results.

### Conclusion

MDD and bipolar disorder are two common mood disorders in psychiatry. Both disorders are associated with severe functional impairment and disability [1-6], but they have different clinical courses, treatment strategies, and prognoses. However, the course of bipolar disorder may begin with depression, and it could be diagnosed as MDD in the initial stage [7,8]. This kind of hidden bipolar disorder may contribute to the treatment resistance observed in unipolar depression [15,29]. Given the therapeutic and prognostic significances of the unipolar-bipolar dichotomy, predicting which patients will show bipolar disorder subsequent to an index diagnosis of MDD is of considerable clinical importance. In our study, the CART method identified five important variables of bipolar disorder conversion. In a simple two- to four-step process, these variables permit the identification of patients with low, intermediate, or high risk for bipolar disorder conversion. The developed model can be applied to routine clinical practice and to facilitate the early diagnosis of bipolar disorder.

## Appendix

Multimedia Appendix 1 Analysis of dataset statistics.

Multimedia Appendix 2 Performance evaluation of the prediction model using the correlation-based feature selection technique (after 12 months).

## Abbreviations

AUC: area under the curve
CART: classiﬁcation and regression tree
CD: ambulatory care expenditure by visit
CFS: correlation-based feature selection
DD: inpatient expenditure by admission
ICD-9-CM: International Classification of Disease, Ninth Revision, Clinical Modification
ID: registry for beneficiaries
LGR: logistic regression
LHID: Longitudinal Health Insurance Database
MDD: major depressive disorder
NHI: National Health Insurance
NHIRD: National Health Insurance Research Database
NHRI: National Health Research Institute
NTD: New Taiwan Dollar
PCA: principal component analysis
RF: random forest
SVM: support vector machine
Weka: Waikato Environment for Knowledge Analysis
