# Supplementary material for: Critical Predictors for the Early Detection of Conversion From Unipolar Major Depressive Disorder to Bipolar Disorder: Nationwide Population-Based Retrospective Cohort Study
Source: JMIR Med Inform. 2020 Apr 3;8(4):e14278. doi: 10.2196/14278 (PMC7165312; doi:10.2196/14278)
Supplement: Multimedia Appendix 1 [file medinform_v8i4e14278_app1.docx]

Multimedia Appendix 1

Analysis of dataset statistics

|  | Converted group, N=398 |  | Non-converted group, N=2284 |  | *P* values |
| --- | --- | --- | --- | --- | --- |
| **Personal characteristics** |  |  |  |  |  |
| Sex |  |  |  |  | 0.067 |
| Male | 141 |  | 922 |  |  |
| Female | 257 |  | 1362 |  |  |
| Age (years) ^a^ | 36 (26 – 48) |  | 38 (26 – 52) |  | 0.003* |
| Follow-up duration (years) ^a^ | 3.43 (1.68 – 6.08) |  | 10.87 (9.87 – 12.10) |  | <0.001* |
| Change of income | 0 (0 – 0) |  | 0 (0 – 0) |  | 0.750 |
| **Severity of disorder** |  |  |  |  |  |
| MDD catastrophic illness | 0 |  | 0 |  | >0.999 |
| Refractory depression | 1 |  | 3 |  | 0.474 |
|  |  |  |  |  |  |
| **Physical comorbidities** |  |  |  |  |  |
| Diseases of respiratory system | 224 |  | 1421 |  | 0.026* |
| Diseases of nervous system and sense organs | 137 |  | 797 |  | 0.909 |
| Diseases of Digestive system | 268 |  | 1436 |  | 0.091 |
| Symptoms, signs, and ill-defined conditions | 204 |  | 1101 |  | 0.277 |
| Diseases of skin and subcutaneous tissue | 109 |  | 605 |  | 0.713 |
| Diseases of genitourinary system | 148 |  | 720 |  | 0.027* |
| Diseases of the musculoskeletal system and connective tissue | 145 |  | 791 |  | 0.494 |
| Diseases of circulatory system | 82 |  | 541 |  | 0.198 |
| Congenital anomalies | 5 |  | 14 |  | 0.186 |
| Endocrine, nutritional and metabolic diseases, and immunity disorders | 69 |  | 398 |  | >0.999 |
| Neoplasms | 37 |  | 197 |  | 0.631 |
| Disease of the blood and blood-forming organs | 21 |  | 71 |  | 0.036* |
| Certain conditions originating in the perinatal period | 0 |  | 3 |  | >0.999 |
| Injury and poisoning | 150 |  | 664 |  | 0.001* |
| Infectious and parasitic diseases | 398 |  | 2284 |  | >0.999 |
| Factors influencing health status and contact with health services | 71 |  | 396 |  | 0.830 |
| External causes of injury of poisoning | 8 |  | 29 |  | 0.243 |
| Coronary artery disease | 13 |  | 113 |  | 0.159 |
| Malignancy | 103 |  | 563 |  | 0.615 |
| Liver cirrhosis | 3 |  | 23 |  | 0.787 |
| Diabetes mellitus | 31 |  | 171 |  | 0.837 |
| Dyslipidemia | 12 |  | 103 |  | 0.226 |
| Hypertension | 46 |  | 319 |  | 0.206 |
| Hyperthyroidism | 4 |  | 31 |  | 0.810 |
| Chronic lung disease | 33 |  | 194 |  | >0.999 |
| Cerebrovascular disease | 10 |  | 86 |  | 0.244 |
| Thyroid disease | 14 |  | 86 |  | 0.887 |
| Other medical comorbidities | 8 |  | 48 |  | >0.999 |
| **Psychiatric comorbidities** |  |  |  |  |  |
| Postpartum depression | 0 |  | 0 |  | >0.999 |
| Anxiety disorder | 135 |  | 556 |  | <0.001* |
| Anxiety states | 115 |  | 500 |  | 0.003* |
| Panic disorder | 18 |  | 111 |  | 0.899 |
| Agoraphobia with panic attacks | 2 |  | 8 |  | 0.650 |
| Generalized anxiety disorder | 16 |  | 105 |  | 0.695 |
| Social phobia | 0 |  | 9 |  | 0.372 |
| Obsessive–compulsive disorders | 18 |  | 32 |  | <0.001* |
| Neurasthenia | 1 |  | 5 |  | >0.999 |
| hypochondriasis | 0 |  | 10 |  | 0.375 |
| Somatization disorder | 3 |  | 4 |  | 0.072 |
| Posttraumatic stress disorder | 10 |  | 16 |  | 0.003* |
| Acute stress disorder | 2 |  | 1 |  | 0.059 |
| Substance use disorder | 12 |  | 47 |  | 0.263 |
| Alcohol misuse and dependence | 12 |  | 47 |  | 0.263 |
| Opioid misuse and dependence | 0 |  | 0 |  | >0.999 |
| Barbiturate misuse and dependence | 0 |  | 0 |  | >0.999 |
| Cocaine misuse and dependence | 0 |  | 0 |  | >0.999 |
| Cannabis misuse and dependence | 0 |  | 0 |  | >0.999 |
| Amphetamine misuse and dependence | 0 |  | 0 |  | >0.999 |
| Hallucinogen misuse and dependence | 0 |  | 0 |  | >0.999 |
| Personality disorder | 28 |  | 70 |  | <0.001* |
| Affective personality disorder | 1 |  | 7 |  | >0.999 |
| Schizoid personality disorder | 0 |  | 2 |  | >0.999 |
| Explosive personality disorder | 0 |  | 0 |  | >0.999 |
| Obsessive-compulsive personality disorder | 3 |  | 8 |  | 0.216 |
| Histrionic personality disorder | 3 |  | 3 |  | 0.046* |
| Dependent personality disorder | 0 |  | 2 |  | >0.999 |
| Antisocial personality disorder | 1 |  | 2 |  | 0.383 |
| Other personality disorders | 18 |  | 39 |  | <0.001* |
| Unspecified personality disorder | 4 |  | 12 |  | 0.280 |
| Attention deficit hyperactivity disorder | 1 |  | 5 |  | >0.999 |
| Sleep disorder | 102 |  | 457 |  | 0.013* |
| Mental retardation | 3 |  | 15 |  | 0.742 |
| Autistic spectrum disorder | 1 |  | 2 |  | 0.383 |
| Eating disorder | 7 |  | 16 |  | 0.068 |
| Polyphagia | 0 |  | 0 |  | >0.999 |
| Anorexia nervosa | 0 |  | 3 |  | >0.999 |
| Eating disorder, unspecified | 4 |  | 5 |  | 0.032* |
| Bulimia nervosa | 4 |  | 7 |  | 0.067 |
| Pica | 0 |  | 0 |  | >0.999 |
| Rumination disorder | 0 |  | 0 |  | >0.999 |
| Psychogenic vomiting | 0 |  | 0 |  | >0.999 |
| Other disorders of eating | 1 |  | 1 |  | 0.275 |
| Inappropriate diet and eating habits | 0 |  | 0 |  | >0.999 |
| Other nonorganic psychoses | 13 |  | 42 |  | 0.081 |
| Disturbance of conduct not elsewhere classified | 1 |  | 5 |  | >0.999 |
|  |  |  |  |  |  |
| **Health care usage behaviors** |  |  |  |  |  |
| Total emergency visits ^a^ | 0 (0 – 1) |  | 0 (0 – 1) |  | 0.006* |
| Total hospitalizations ^a^ | 0 (0 – 1) |  | 0 (0 – 1) |  | 0.195 |
| Total outpatient visits ^a^ | 15 (10 – 22) |  | 13 (8 – 19) |  | <0.001* |
| Total psychiatric outpatient visits ^a^ | 7 (3 – 10) |  | 4 (1 – 7) |  | <0.001* |
| Total psychiatric hospitalizations ^a^ | 0 (0 – 0) |  | 0 (0 – 0) |  | <0.001* |
| Emergency visits - January ^a^ | 0 (0 – 0) |  | 0 (0 – 0) |  | 0.292 |
| Emergency visits - February ^a^ | 0 (0 – 0) |  | 0 (0 – 0) |  | 0.188 |
| Emergency visits - March ^a^ | 0 (0 – 0) |  | 0 (0 – 0) |  | 0.461 |
| Emergency visits - April ^a^ | 0 (0 – 0) |  | 0 (0 – 0) |  | 0.232 |
| Emergency visits - May ^a^ | 0 (0 – 0) |  | 0 (0 – 0) |  | 0.269 |
| Emergency visits - June ^a^ | 0 (0 – 0) |  | 0 (0 – 0) |  | 0.007* |
| Emergency visits - July ^a^ | 0 (0 – 0) |  | 0 (0 – 0) |  | 0.470 |
| Emergency visits - August ^a^ | 0 (0 – 0) |  | 0 (0 – 0) |  | 0.219 |
| Emergency visits - September ^a^ | 0 (0 – 0) |  | 0 (0 – 0) |  | 0.196 |
| Emergency visits - October ^a^ | 0 (0 – 0) |  | 0 (0 – 0) |  | 0.836 |
| Emergency visits - November ^a^ | 0 (0 – 0) |  | 0 (0 – 0) |  | 0.285 |
| Emergency visits - December ^a^ | 0 (0 – 0) |  | 0 (0 – 0) |  | 0.086 |
| Hospitalizations - January ^a^ | 0 (0 – 0) |  | 0 (0 – 0) |  | 0.766 |
| Hospitalizations - February ^a^ | 0 (0 – 0) |  | 0 (0 – 0) |  | 0.744 |
| Hospitalizations - March ^a^ | 0 (0 – 0) |  | 0 (0 – 0) |  | 0.812 |
| Hospitalizations - April ^a^ | 0 (0 – 0) |  | 0 (0 – 0) |  | 0.171 |
| Hospitalizations - May ^a^ | 0 (0 – 0) |  | 0 (0 – 0) |  | 0.490 |
| Hospitalizations - June ^a^ | 0 (0 – 0) |  | 0 (0 – 0) |  | 0.122 |
| Hospitalizations - July ^a^ | 0 (0 – 0) |  | 0 (0 – 0) |  | 0.977 |
| Hospitalizations - August ^a^ | 0 (0 – 0) |  | 0 (0 – 0) |  | 0.175 |
| Hospitalizations - September ^a^ | 0 (0 – 0) |  | 0 (0 – 0) |  | 0.733 |
| Hospitalizations - October ^a^ | 0 (0 – 0) |  | 0 (0 – 0) |  | 0.940 |
| Hospitalizations - November ^a^ | 0 (0 – 0) |  | 0 (0 – 0) |  | 0.224 |
| Hospitalizations - December ^a^ | 0 (0 – 0) |  | 0 (0 – 0) |  | 0.668 |
| Outpatient visits - January ^a^ | 0 (0 – 2) |  | 0 (0 – 2) |  | 0.086 |
| Outpatient visits - February ^a^ | 0 (0 – 2) |  | 0 (0 – 2) |  | 0.067 |
| Outpatient visits - March ^a^ | 0 (0 – 2) |  | 0 (0 – 2) |  | 0.108 |
| Outpatient visits - April ^a^ | 0 (0 – 2) |  | 0 (0 – 2) |  | 0.077 |
| Outpatient visits - May ^a^ | 0 (0 – 2) |  | 0 (0 – 2) |  | 0.074 |
| Outpatient visits - June ^a^ | 0 (0 – 2) |  | 0 (0 – 2) |  | 0.009* |
| Outpatient visits - July ^a^ | 0 (0 – 2) |  | 0 (0 – 2) |  | 0.055 |
| Outpatient visits - August ^a^ | 0 (0 – 2) |  | 0 (0 – 2) |  | 0.117 |
| Outpatient visits - September ^a^ | 0 (0 – 2) |  | 0 (0 – 2) |  | 0.071 |
| Outpatient visits - October ^a^ | 1 (0 – 2.25) |  | 0 (0 – 2) |  | 0.074 |
| Outpatient visits - November ^a^ | 1 (0 – 2) |  | 0 (0 – 2) |  | 0.016* |
| Outpatient visits - December ^a^ | 0 (0 – 2) |  | 0 (0 – 2) |  | 0.142 |
| Psychiatric outpatient visits - January ^a^ | 0 (0 – 1) |  | 0 (0 – 1) |  | 0.003* |
| Psychiatric outpatient visits - February ^a^ | 0 (0 – 1) |  | 0 (0 – 0) |  | 0.006* |
| Psychiatric outpatient visits - March ^a^ | 0 (0 – 1) |  | 0 (0 – 0) |  | <0.001* |
| Psychiatric outpatient visits - April ^a^ | 0 (0 – 1) |  | 0 (0 – 1) |  | 0.011* |
| Psychiatric outpatient visits - May ^a^ | 0 (0 – 1) |  | 0 (0 – 1) |  | <0.001* |
| Psychiatric outpatient visits - June ^a^ | 0 (0 – 1) |  | 0 (0 – 0) |  | <0.001* |
| Psychiatric outpatient visits - July ^a^ | 0 (0 – 1) |  | 0 (0 – 1) |  | <0.001* |
| Psychiatric outpatient visits - August ^a^ | 0 (0 – 1) |  | 0 (0 – 1) |  | 0.004* |
| Psychiatric outpatient visits - September ^a^ | 0 (0 – 1) |  | 0 (0 – 1) |  | 0.009* |
| Psychiatric outpatient visits - October ^a^ | 0 (0 – 1) |  | 0 (0 – 1) |  | 0.005* |
| Psychiatric outpatient visits - November ^a^ | 0 (0 – 1) |  | 0 (0 – 1) |  | <0.001* |
| Psychiatric outpatient visits - December ^a^ | 0 (0 – 1) |  | 0 (0 – 1) |  | 0.001* |
| Psychiatric hospitalizations - January ^a^ | 0 (0 – 0) |  | 0 (0 – 0) |  | 0.082 |
| Psychiatric hospitalizations - February ^a^ | 0 (0 – 0) |  | 0 (0 – 0) |  | 0.187 |
| Psychiatric hospitalizations - March ^a^ | 0 (0 – 0) |  | 0 (0 – 0) |  | 0.612 |
| Psychiatric hospitalizations - April ^a^ | 0 (0 – 0) |  | 0 (0 – 0) |  | 0.104 |
| Psychiatric hospitalizations - May ^a^ | 0 (0 – 0) |  | 0 (0 – 0) |  | 0.104 |
| Psychiatric hospitalizations - June ^a^ | 0 (0 – 0) |  | 0 (0 – 0) |  | 0.068 |
| Psychiatric hospitalizations - July ^a^ | 0 (0 – 0) |  | 0 (0 – 0) |  | 0.248 |
| Psychiatric hospitalizations - August ^a^ | 0 (0 – 0) |  | 0 (0 – 0) |  | 0.060 |
| Psychiatric hospitalizations - September ^a^ | 0 (0 – 0) |  | 0 (0 – 0) |  | 0.141 |
| Psychiatric hospitalizations - October ^a^ | 0 (0 – 0) |  | 0 (0 – 0) |  | 0.604 |
| Psychiatric hospitalizations - November ^a^ | 0 (0 – 0) |  | 0 (0 – 0) |  | 0.767 |
| Psychiatric hospitalizations - December ^a^ | 0 (0 – 0) |  | 0 (0 – 0) |  | 0.247 |
| Emergency visits - Spring ^a^ | 0 (0 – 0) |  | 0 (0 – 0) |  | 0.200 |
| Emergency visits - Summer ^a^ | 0 (0 – 0) |  | 0 (0 – 0) |  | 0.020* |
| Emergency visits - Fall ^a^ | 0 (0 – 0) |  | 0 (0 – 0) |  | 0.212 |
| Emergency visits - Winter ^a^ | 0 (0 – 0) |  | 0 (0 – 0) |  | 0.055 |
| Hospitalizations - Spring ^a^ | 0 (0 – 0) |  | 0 (0 – 0) |  | 0.187 |
| Hospitalizations - Summer ^a^ | 0 (0 – 0) |  | 0 (0 – 0) |  | 0.099 |
| Hospitalizations - Fall ^a^ | 0 (0 – 0) |  | 0 (0 – 0) |  | 0.614 |
| Hospitalizations - Winter ^a^ | 0 (0 – 0) |  | 0 (0 – 0) |  | 0.796 |
| Outpatient visits - Spring ^a^ | 2 (0 – 6) |  | 2 (0 – 5) |  | 0.038* |
| Outpatient visits - Summer ^a^ | 2 (0 – 7) |  | 2 (0 – 5 |  | 0.012* |
| Outpatient visits - Fall ^a^ | 3 (0 – 7) |  | 2 (0 – 6) |  | 0.013* |
| Outpatient visits - Winter ^a^ | 2 (0 – 7) |  | 2 (0 – 6) |  | 0.047* |
| Psychiatric outpatient visits - Spring ^a^ | 1 (0 – 3) |  | 0 (0 – 2) |  | <0.001* |
| Psychiatric outpatient visits - Summer ^a^ | 1 (0 – 3) |  | 0 (0 – 2) |  | <0.001* |
| Psychiatric outpatient visits - Fall ^a^ | 1 (0 – 3) |  | 0 (0 – 2) |  | <0.001* |
| Psychiatric outpatient visits - Winter ^a^ | 1 (0 – 3) |  | 0 (0 – 2) |  | <0.001* |
| Psychiatric hospitalizations - Spring ^a^ | 0 (0 – 0) |  | 0 (0 – 0) |  | 0.034* |
| Psychiatric hospitalizations - Summer ^a^ | 0 (0 – 0) |  | 0 (0 – 0) |  | 0.012* |
| Psychiatric hospitalizations - Fall ^a^ | 0 (0 – 0) |  | 0 (0 – 0) |  | 0.286 |
| Psychiatric hospitalizations - Winter ^a^ | 0 (0 – 0) |  | 0 (0 – 0) |  | 0.026* |
|  |  |  |  |  |  |
| **Use of psychotropics** |  |  |  |  |  |
| Antidepressant use | 121 |  | 593 |  | 0.066 |
| Antipsychotics use | 101 |  | 338 |  | <0.001* |
| Benzodiazepine use | 109 |  | 641 |  | 0.809 |
| Mood stabilizer use | 58 |  | 130 |  | <0.001* |
| Maximum kinds of antidepressant use in one visit ^a^ | 0 (0 – 1) |  | 0 (0 – 1) |  | 0.011* |
| Maximum kinds of antipsychotics use in one visit ^a^ | 0 (0 – 1) |  | 0 (0 – 0) |  | <0.001* |
| Maximum kinds of benzodiazepine use in one visit ^a^ | 0 (0 – 2) |  | 0 (0 – 1) |  | 0.003* |
| Maximum kinds of mood stabilizer use in one visit ^a^ | 0 (0 – 0) |  | 0 (0 – 0) |  | <0.001* |
| Kinds of antidepressant use within first six months of enrollment ^a^ | 0 (0 – 2) |  | 0 (0 – 1) |  | <0.001* |
| Kinds of antipsychotics use within first six months of enrollment ^a^ | 0 (0 – 1) |  | 0 (0 – 0) |  | <0.001* |
| Kinds of benzodiazepine use within first six months of enrollment ^a^ | 0 (0 – 4) |  | 0 (0 – 1) |  | <0.001* |
| Kinds of mood stabilizer use within first six months of enrollment ^a^ | 0 (0 – 0) |  | 0 (0 – 0) |  | <0.001* |

^a^ Median (interquartile range); * Statistical significance
