# Supplementary material for: Critical Predictors for the Early Detection of Conversion From Unipolar Major Depressive Disorder to Bipolar Disorder: Nationwide Population-Based Retrospective Cohort Study
Source: JMIR Med Inform. 2020 Apr 3;8(4):e14278. doi: 10.2196/14278 (PMC7165312; doi:10.2196/14278)
Supplement: Multimedia Appendix 2 [file medinform_v8i4e14278_app2.docx]

Multimedia Appendix 2

Performance evaluation of prediction model using CFS feature selection technique (after 12 months)

| Dataset | Method |  | Metrics | | | |
| --- | --- | --- | --- | --- | --- | --- |
|  |  |  | ACC | SEN | SPE | AUC |
| Training/Validation  (10-fold cross validation) | C4.5 |  | 0.604 | 0.551 | 0.658 | 0.633 |
|  | SVM |  | 0.524 | 0.524 | 0.524 | 0.586 |
|  | RF |  | 0.649 | 0.653 | 0.644 | 0.695 |
|  | LGR |  | 0.660 | 0.547 | 0.773 | 0.728 |

LGR logistic regression; RF random forest; SVM support vector machine; ACC Accuracy; SEN Sensitivity; SPE Specificity; AUC Area under the curve
